# Non‐Volatile In_3_SbTe_2_ Multispectral Responsive Emitter With Visible Color and Infrared Display‐Erasure Function

**DOI:** 10.1002/nap2.70084

**Published:** 2026-04-16

**Authors:** Jinlong Huang, Xiuyu Chen, Shuwen Zheng, Kun Yu, Mengdan Qian, Yufang Liu

**Affiliations:** ^1^ Henan Key Laboratory of Infrared Spectrum Measures and Applications, School of Optoelectronic Engineering Henan Normal University Xinxiang China

**Keywords:** multispectral, phase materials, spectral selectivity, switchable, wide‐angle insensitivity

## Abstract

With the growing integration of wide‐spectrum detection technology, metamaterials featuring multispectrum dynamic response have emerged as an effective approach to enhance both target camouflage and noncamouflage. However, conventional response materials typically exhibit only a single‐band or static spectral response, which restricts their capacity to adapt to complex environments. Herein, a novel multispectral response emitter (MRE) composed of Al/Ge/SiO_2_/IST/ZnS is fabricated using a thin‐film evaporation deposition system, enabling visible color modulation and high/low infrared emissivity switching. The MRE can precisely match the colors of various environments, including the blue sky, ocean, yellow grassland, and green leaves, through the utilization of the thin‐film interference effect within the visible band. Meanwhile, by the rational control of the phase transition state of the IST, it can switch between high and low emissivity within two atmospheric windows (MWIR and LWIR), achieving the “display‐erasure” of infrared image. Leveraging the dual‐band collaborative response characteristics, the feasibility of encoding multilayered information on the MRE is experimentally verified. Furthermore, the MRE exhibits wide‐angle insensitivity, maintaining consistent performance over a broad range of observation angles. This work introduces new approaches for multiband dynamic spectral response and holds significant application potential, such as information encryption and thermal camouflage.

## Graphical abstract

A multispectral response emitter based on In_3_SbTe_2_ is proposed for multispectral information encryption and dynamic infrared camouflage. It enables independent control of the switching between visible color and infrared emissivity, realizes temperature‐triggered infrared pattern reconstruction, and achieves complete concealment in the visible band. This emitter is applicable to infrared stealth anticounterfeiting and secure optical information storage.

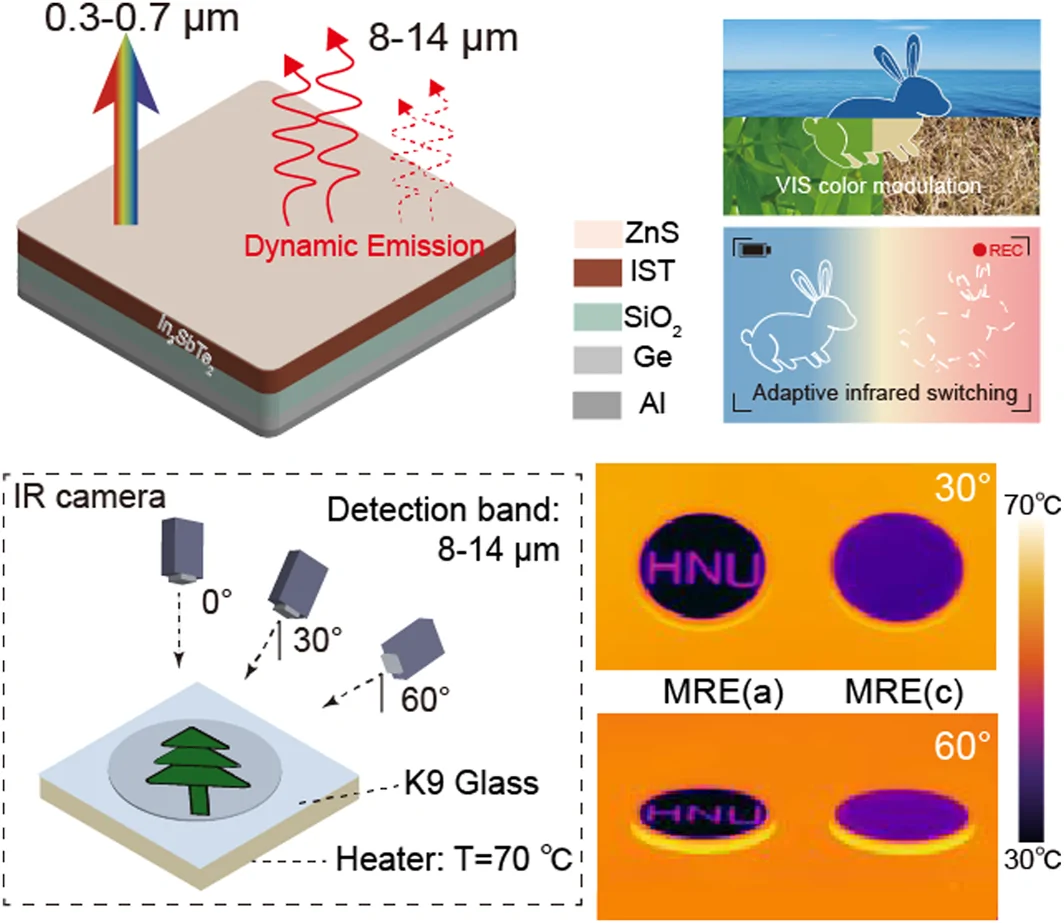

## 1 Introduction

Emerging metamaterials can respond to external stimuli and regulate electromagnetic waves, thereby finding extensive applications in many important fields, including camouflage [1, 2, 3], information security [4, 5, 6], thermal management [7, 8, 9], energy harvesting [10, 11], and high‐performance sensing systems [12, 13]. With the development of detection technology toward multiband compatibility, the traditional single‐band and static response materials can no longer handle complex environments [14, 15, 16, 17]. Therefore, this highlights the urgent necessity for dynamic multispectral response metamaterials. Due to the distinct detection principles of various frequency bands, the requirements for modulating the corresponding electromagnetic waves also vary. Specifically, precise matching with various environmental colors is required to realize visual response in the visible (VIS) spectrum. Regarding the infrared band, the identification and camouflage of targets primarily rely on the emission characteristics of the atmospheric windows (3–5 μm and 8–14 μm). Furthermore, the integration of multiple response features into single system can significantly enhance antireplication capabilities [4, 18, 19]. The multiresponsive metamaterial enables encoding of one information channel via specific visible colors (e.g., yellow, blue, and green), while dynamically switching between high and low emissivity within the infrared atmospheric window to encode a second information channel. This approach not only enhances the information encoding capacity but also significantly improves the information security level in anticounterfeiting technologies.

Metamaterials, a novel type of artificial composite materials with extraordinary electromagnetic/optical properties, have risen to become a highly promising approach for thermal radiation modulation owing to their unique functionalities and precise control capabilities over the wavelength [20, 21], amplitude [6, 22], propagation direction [23, 24], and polarization state [25] of electromagnetic waves. However, a significant challenge exists in the design of a metamaterial that can achieve precise visible color modulation and autonomous switching of the infrared emissivity characteristics. Traditional materials face substantial limitations as they are inherently static and lack the adaptability required for complex thermal environments [9, 26, 27, 28]. Consequently, the development of dynamic control response technologies is urgently needed to tackle these challenges. Although dynamic response technologies have been comprehensively investigated through methods, such as thermochromic [29, 30, 31], electrophoretic [32, 33], and mechanically driven devices [34, 35], the majority of these systems demand a continuous external energy supply. This dependence leads to significant limitations in terms of practical applicability. Recently, Zhou et al. proposed a programmable color thermal camouflage strategy based on the phase‐change material IST [36]. Huang et al. utilized the phase‐change material IST to realize switchable visible and infrared camouflage with effective thermal management [37]. Zha et al. achieved high‐contrast emissivity switching across the entire long‐wave infrared band using the phase‐change material IST [38]. Based on the above progress, the feasibility of employing IST for switchable infrared spectral modulation has been well demonstrated. However, the simultaneous and facile realization of visible information encoding, dynamic infrared information encoding, and visible‐infrared (VIS‐IR) switchable camouflage remains a critical challenge.

Here, we have successfully designed and fabricated a novel multispectral responsive emitter (MRE), which integrates visible color modulation with high/low infrared emissivity switching capabilities. The emitter features a multilayer film structure consisting of Al/Ge/SiO_2_/IST/ZnS from bottom to top. The main advantages of the emitter are as follows: (i) Dynamic Infrared Emissivity Switching: By leveraging the tunable characteristics of the IST film, the MRE demonstrates high emissivity (*λ*_4.17 μm_ ∼ 0.86 and *λ*_9.36 μm_ ∼ 0.99) in both the mid‐wave infrared (MWIR, *ε*_3–5 μm_ ∼ 0.24) and long‐wave infrared (LWIR, *ε*_8–14 μm_ ∼ 0.53) bands. Moreover, it can be dynamically switched to a low infrared emissivity state (*ε*_3–14 μm_ ∼ 0.21). (ii) Precise Color Modulation: Through the precise regulation of the visible reflection spectrum, the emitter is capable of dynamically adjusting its appearance color across various operating environments. (iii) Wide‐Angle Insensitivity: Within an observation angle range of 0°–60°, the switching infrared characteristic behavior of the emitter can be clearly observed via the LWIR camera. (iv) Simple and Large‐Scale Fabrication: The emitter adopts a straightforward multilayer film stacking design, which is suitable for large‐scale fabrication and lowers the technical barriers for practical implementation. Finally, we systematically evaluate the encoding information capacity of the MRE, demonstrating its stability in dynamic switching for encoding information at large observation angles. The novel MRE featuring dual‐band and tunable advantages presents a promising approach for the next‐generation multispectral dynamic regulation system.

## 2 Results and Discussion

### 2.1 Design Principles of MRE

Figure 1a presents the MRE that enables adaptive switching between the visible and infrared bands. As a nonvolatile phase‐change material (PCM), IST exhibits phase‐transition characteristics that are markedly different from those of PCMs. Generally, PCMs experience a phase transformation from an amorphous state to a crystalline state, along with a change from a high‐refractive‐index dielectric material (*n*_*a*_ > *k*_*a*_) to an even higher‐refractive‐index dielectric material (*n*_*c*_ > *k*_*c*_). In contrast, the phase transition of IST entails a conversion from a lossless dielectric to a lossy metallic in Supporting Information S1: Figure S1 [38], whose optical properties remain stable over long‐term periods without the necessity of continuous energy input. This unique property suggests that adaptive regulation of infrared emissivity can be effectively accomplished via controlling the crystalline state of the IST film. As shown in Figure 1b, this MRE is capable of independently modulating both the visible color and dynamically switching the infrared emissivity. This advantage enables a single device to be customized for various application scenarios. In dual‐band camouflage applications, the visible color modulation of the MRE can be precisely matched to various operating environments (including ocean, green leaves, and yellow grass) by mimicking the color characteristics of the target background. This approach not only achieves visual camouflage but also enables visible encoding information. By utilizing the infrared switching capability of the IST film, the MRE is able to dynamically modulate its infrared radiation properties according to different operational scenarios.

**FIGURE 1:**
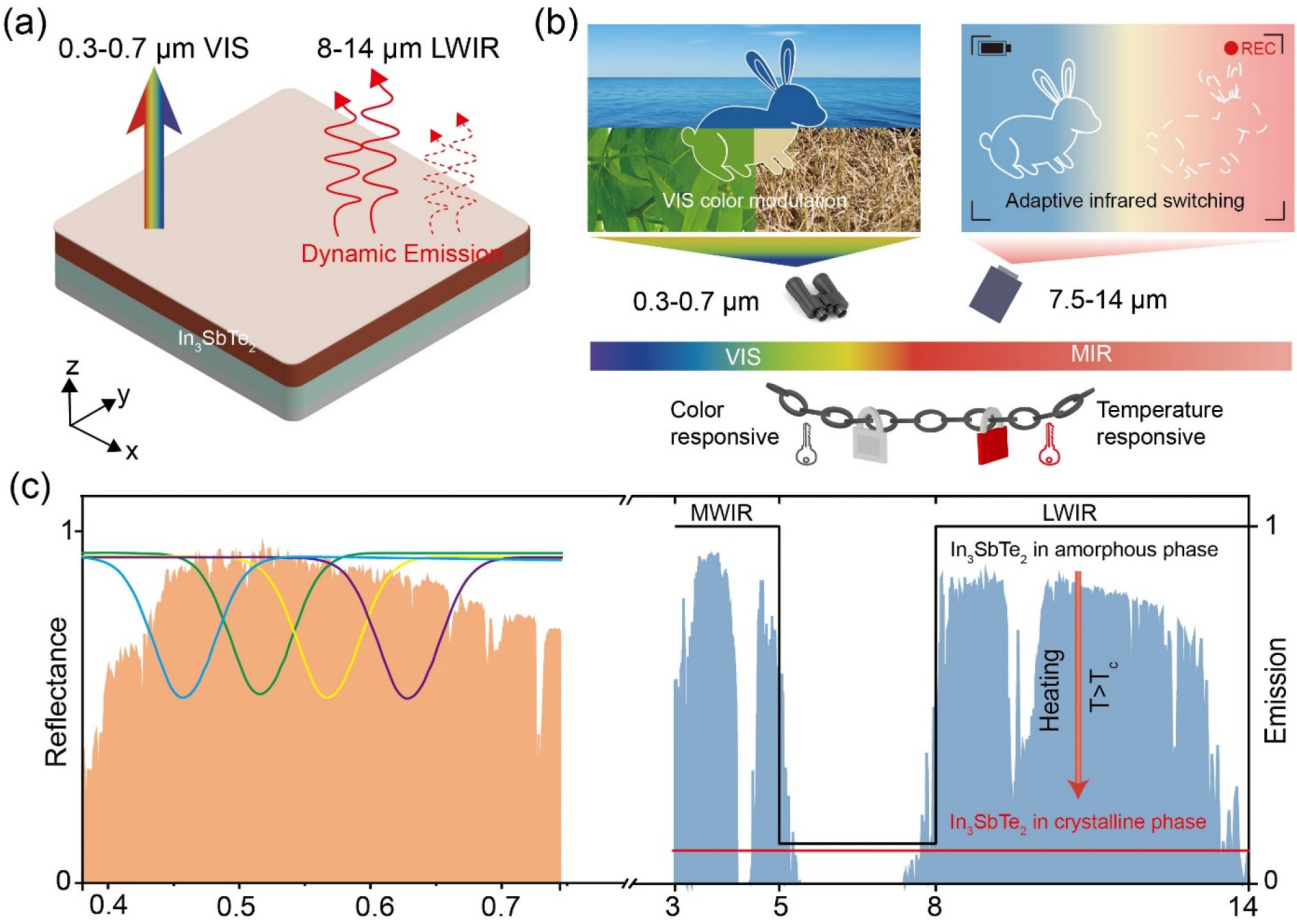
Design principles and applications of the MRE. (a) Schematic illustration of the dynamic color/emissivity switching thermal radiation of the MRE. (b) Dual‐band application demonstration of the MRE: visible color modulation and infrared “display‐erasure”. (c) The ideal response spectrum of the target MRE in amorphous and crystalline states.

Generally, this multifunctional responsive structure is realized through the rational design of its spectral selective radiation across different frequency bands. Figure 1c illustrates the ideal spectral response of the target MRE in the VIS‐IR band. To fulfill the requirements of dual‐band spectral control, the MRE must perform distinct functions across different spectral regions. In the visible band, it is necessary for the MRE to display a variety of colors corresponding to diverse operating environments. In the infrared spectrum, the infrared emissivity properties are determined by the crystalline state of the IST film. For the IST film in an amorphous state, the MRE(a) shows high emissivity in both the MWIR and LWIR bands, enabling the effective detection of targets. Upon transitioning to the crystalline state, infrared radiation from MRE(c) is significantly attenuated within the IST film, leading to low emissivity across the entire infrared region. This switchable characteristic of the IST film allows the MRE to achieve selective modulation of the infrared spectrum.

### 2.2 Structural Design and Mechanism Analysis of MRE

Figure 2a illustrates the structural schematic of the MRE. Structurally, the MRE consists of five distinct material layers arranged in the following order from bottom to top: Al, Ge, SiO_2_, IST, and ZnS. The corresponding thicknesses of these layers are 200 nm, 320 nm, 250 nm, 220 nm, and 230 nm, respectively. Among these layers, the top ZnS layer is chosen due to its high‐value refractive index within the VIS spectrum [39]. By leveraging its thin‐film interference properties, precise modulation of visible colors is achieved. In essence, the visible structural color is predominantly determined by the thickness of the top ZnS layer. Meanwhile, the design also utilizes the phase‐change properties of the IST film. This material is able to convert from an amorphous to a crystallized state at the critical transition temperature of 300°C. The photonic resonance structure of the MRE further strengthens the modulation of the emissivity induced by the phase transition process. Furthermore, the dielectric constant parameters of Al, Ge, and SiO_2_ utilized in this study are obtained from references [39, 40, 41]. To evaluate the performance of the MRE, COMSOL Multiphysics software is utilized for numerical simulation of its absorption and electromagnetic field distribution characteristics. Figure 2b presents the simulated infrared emissivity spectrum of the MRE. For the IST film in an amorphous state, the MRE(a) shows resonance peaks with intensities of 0.62 and 0.99 at wavelengths of 4.17 and 9.36 μm, respectively. The corresponding average emissivity in the MWIR and LWIR regions are 0.16 and 0.35, respectively. When the IST film transitions to the crystalline state, it exhibits properties analogous to those of a lossy metal, which significantly suppresses thermal emission across the entire infrared band. Consequently, the MRE(c) exhibits a low emissivity (*ε*_3–14 μm_ ∼ 0.11) throughout the infrared region.

**FIGURE 2:**
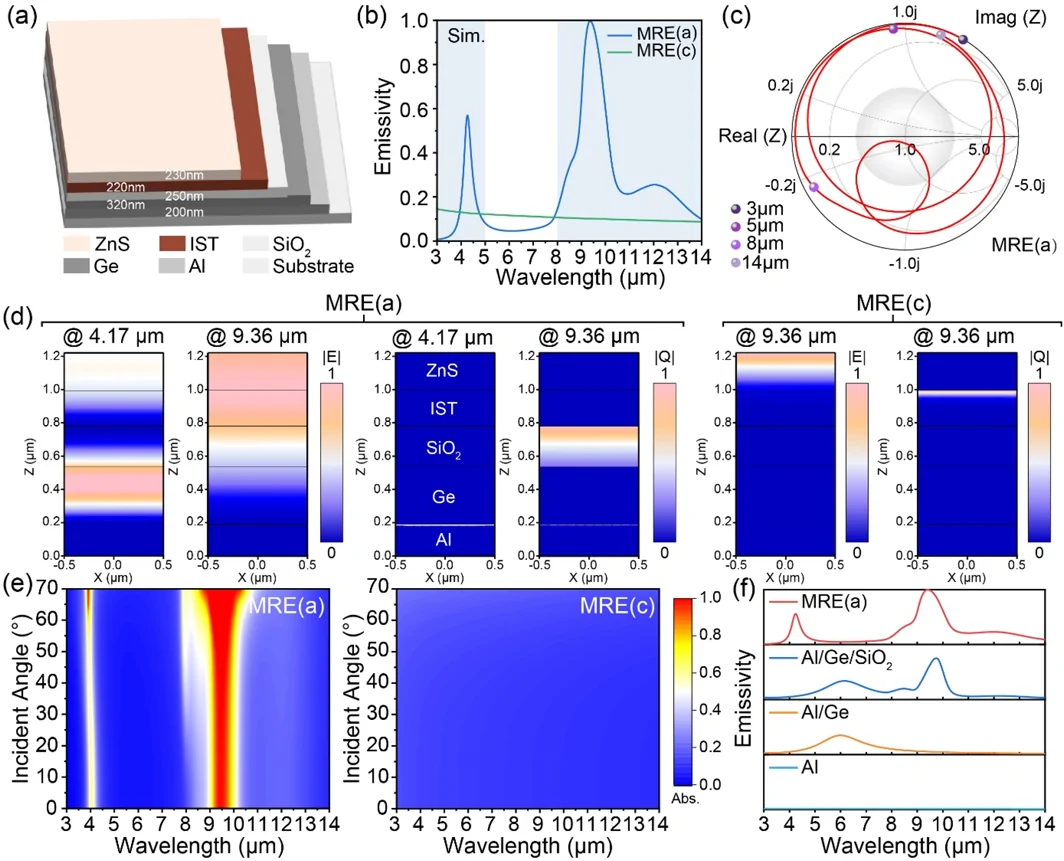
The mechanisms of MRE's dynamic infrared emissivity regulation. (a) The structural diagram of MRE. (b) Simulated infrared emissivity spectrum of the MRE (amorphous and crystalline states). (c) Calculated normalized impedance of MRE(a). (d) The electric field density and power loss density distributions of the MRE at wavelengths of 4.17 and 9.36 μm, respectively. (e) The simulated absorption spectra of MRE (amorphous and crystalline states) at incident angles ranging from 0° to 70°. (f) The analyses of the emissivity spectrum for different layer combinations.

To deeply analyze the mechanism of the resonance peaks, we incorporate the impedance matching theory and carry out a systematic investigation via the visual characterization of electric field density and power loss density distributions. According to the impedance matching theory, the degree to which the normalized impedance of an object (*Z* = *Z*_re_ + *Z*_imj_) matches the impedance of air (*Z* = 1 + 0j) within a specific frequency band directly dictates its reflectivity. Specifically, when *Z*_re_ approaches 1 and *Z*_im_ approaches 0, the reflectivity reaches its minimum value. Figure 2c presents the normalized impedance distribution of the MRE(a). The center and boundary of the gray circular shaded area represent the impedance values corresponding to emissivity values of 1 and 0.8, respectively. As the wavelength increases, it can be clearly observed that the impedance within the LWIR band converges toward the central gray‐shaded area. This phenomenon shows that MRE(a) achieves high emissivity within the LWIR band, which corresponds closely to the previous simulation results. To clarify the underlying physical mechanisms of the resonance peaks, Figure 2d presents the electric field density and power loss density distributions characteristics of MRE(a) at wavelengths of 4.17 and 9.36 μm. For the IST film in an amorphous state, it remains transparent to infrared radiation. The 4.17 μm resonance peak is mainly localized in the Ge layer, whereas the one at 9.36 μm results from structural resonance with the collective contributions of all dielectric layers (including Ge, SiO_2_, IST, and ZnS). The visualized power loss density distributions of MRE(a) demonstrate that the bulk of incident wave energy is primarily dissipated in the bottom metallic Al layer at the resonance wavelengths of 4.17 μm. At another resonance wavelength of 9.36 μm, energy loss exhibits predominant localization within the SiO_2_ layer. This is because the imaginary dielectric constant of SiO_2_ reaches a local maximum in the LWIR band, leading it to exhibit lossy metal‐like characteristics (*n* < *k*). When the IST layer undergoes a transition to the crystalline state, the power loss distribution at 9.36 μm reveals that the bulk of incident wave energy is subject to dominant dissipation within the IST layer. This behavior can be more intuitively visualized from the normalized electric field and power loss in Supporting Information S1: Figure S2. This finding further validates that the crystalline IST film demonstrates metallic‐like behavior and can effectively suppress infrared thermal emission.

To investigate the angle insensitivity of the MRE and evaluate the functional significance of each film layer, the influence of altered incident angles as well as film layer configurations on infrared band characteristics are simulated. Figure 2e illustrates the impact associated with incident angle modifications on the MRE's absorption characteristics. Even when the incident angle ranges from 0° to 70°, the absorption spectrum of MRE(a) at the resonance wavelengths of 4.17 and 9.36 μm exhibits negligible changes. For the IST film in the crystalline state, it exhibits metallic‐like properties. Under these conditions, the absorption spectrum of MRE(c) remains consistent across different incident angles and wavelengths. This finding demonstrates that the MRE possesses excellent angle‐insensitivity performance within a wide detection range. To further clarify the functions and necessity of different constituent layers in the MRE, Figure 2f shows simulations and comparative analyses of the emissivity spectra for different layer combinations. When only the bottom metallic Al layer exists, the emitter exhibits a low emissivity. The Al layer acts as a highly reflective metal substrate, providing strong optical confinement within the multilayer structure. After constructing the Al/Ge structure, a weak resonance peak at 6.01 μm is observed, which can be attributed to the standing‐wave effect. This effect arises because the thickness of the Ge layer is close to *λ*/4*n*, where *λ* denotes the resonant wavelength and *n* is the refractive index of the dielectric material. Upon adding the SiO_2_ layer to form the Al/Ge/SiO_2_ structure, the original resonance at 6.01 μm is well preserved, whereas an additional strong resonance peak emerges at 9.5 μm. This peak at 9.5 μm originates from the intrinsic optical loss in SiO_2_ within the long‐wave infrared region, where its imaginary part of the dielectric constant reaches a local maximum (*n* < *k*). In the case of MRE(a), the incorporation of the IST and ZnS layers not only causes the resonance peaks to shift to 4.17 and 9.36 μm but also significantly enhances the intensities of these resonance peaks. Nevertheless, it exerts nearly no influence on the low‐emissivity state of the MRE(c). Through making reasonable selections of the different layers of MRE and optimizing the parameters, the MRE can precisely modulate its switchable selective radiation function.

### 2.3 Material Characterization

The IST film is fabricated using magnetron sputtering, and its performance and structural characteristics are validated through various characterization techniques. Figure 3a presents the X‐ray diffraction (XRD) pattern of the IST film. The lack of distinct diffraction peaks in the unannealed IST film suggests its amorphous state. In contrast, after annealing at 300°C for 10 min, the IST film displays four characteristic diffraction peaks‐(2 0 0) (2 2 0), (2 2 2), and (4 2 0), which are associated with the crystalline state of IST [42, 43]. These results confirm that the thermal treatment effectively induces an amorphous to crystalline phase transition in the IST film. To further explore the impact of annealing on the IST film surfaces, the surface roughness characteristics of the IST film before and after annealing is characterized using atomic force microscopy (AFM) in Figure 3b,c. The experimental results show that the surface roughness of the IST film increases slightly from 0.18 to 0.38 nm after annealing, indicating minimal alterations. This finding reveals that thermal annealing has negligible effect on the flatness of the IST film, thus providing a robust basis for subsequent thin‐film deposition via evaporation techniques.

**FIGURE 3:**
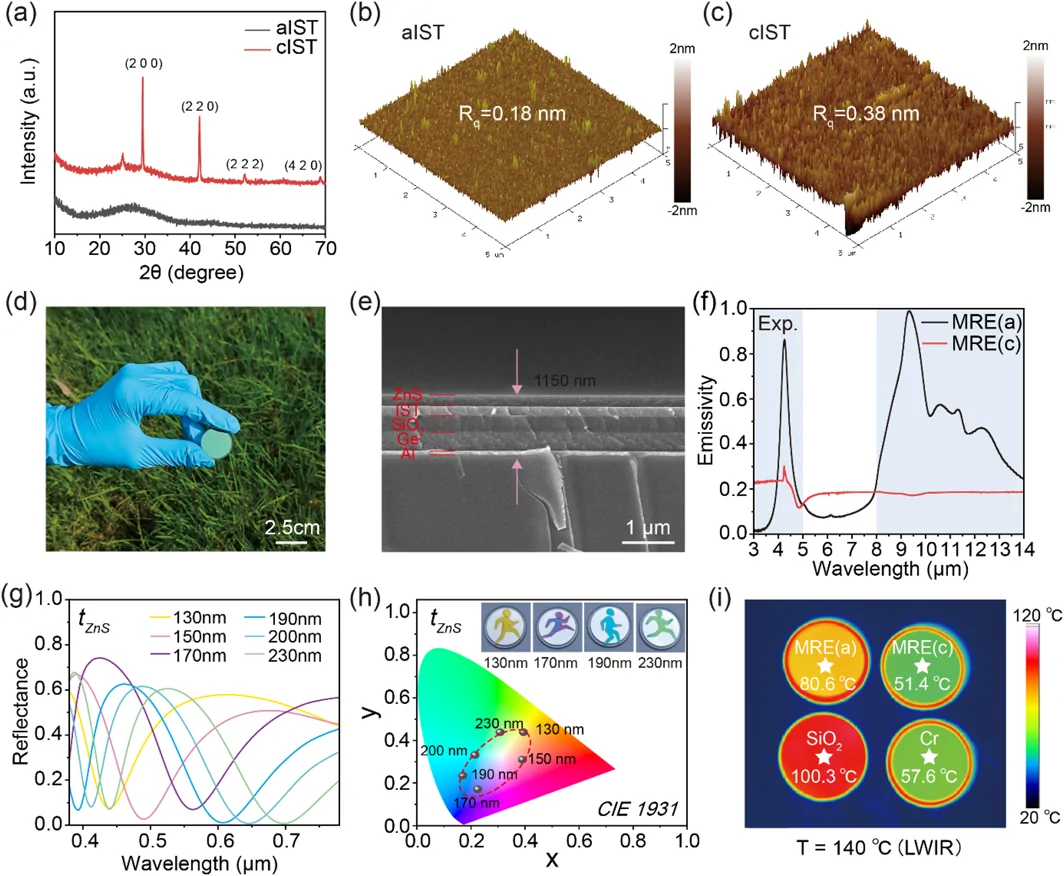
Characterization of the phase transition properties of the IST film and the optical properties of the fabricated MRE. (a) The XRD patterns of IST film (amorphous and crystalline states). (b, c) The atomic force microscope (AFM) surface morphology of the IST film before and after annealing. (d) Photograph of the fabricated MRE. (e) The SEM cross‐sectional image of the MRE. (f) Measured infrared emissivity spectrum of the MRE (amorphous and crystalline states). (g) Visible reflection spectra of the fabricated MRE with different ZnS layer thicknesses. (h) CIE‐1931 color coordinate images for ZnS layers of varying thicknesses (i) LWIR images of the MRE (amorphous and crystalline states) and the reference objects (*T* = 140°C).

Additionally, the fabrication and characterization of the MRE are successfully accomplished. The fabrication process of the MRE involves the integration of multiple thin‐film deposition techniques. With SiO_2_ material serving as the substrate, Al, Ge, and SiO_2_ films are sequentially deposited through electron beam evaporation. Subsequently, the IST phase‐change layer is deposited onto this structure using magnetron sputtering. Finally, the ZnS film is deposited by electron beam evaporation to finalize the device fabrication. Among them, the fabricated MRE is annealed in a tube furnace at 300°C for 10 min under vacuum to obtain its crystalline phase. As depicted in Figure 3d, a photograph of the fabricated MRE is presented. The visible coloration imparted to the MRE can be used not only to prevent visual tracking but also for visible encoding information. Figure 3e presents the cross‐sectional image of the MRE observed by scanning electron microscopy (SEM). The results indicate that the thicknesses of the Al, Ge, SiO_2_, IST, and ZnS layers in the MRE are approximately 200 nm, 320 nm, 250 nm, 220 nm, and 230 nm, respectively. The thickness of the MRE cross‐section is further characterized through the selection of different scales and EDS elemental mapping characterization in Supporting Information S1: Figures S4 and S5. This series of characterization results verifies the stability of the MRE fabrication process and the precision of the structural design, thus providing a solid structural foundation for subsequent device performance evaluation.

### 2.4 Optical Characterization

The infrared emissivity spectrum of MRE is closely associated with the crystalline state of the IST layer due to its phase transition properties. To investigate the tunable emissivity of the MRE, its infrared spectral properties are characterized by utilizing a Fourier transform infrared (FTIR) spectrometer. Figure 3f depicts the measured emissivity spectrum of the MRE under different IST states. For the IST film in the amorphous state, the MRE(a) shows distinct resonance peaks at wavelengths of 4.17 and 9.36 μm, with high emissivity values of 0.86 and 0.99, respectively. The corresponding average emissivity in the MWIR and LWIR regions are 0.24 and 0.53, respectively. This is effective for capturing infrared images. The high temperature will induce the phase transition in the IST film, leading to its transition from an amorphous state to a crystalline state. Meanwhile, the MRE(c) displays a low emissivity state (*ε*_3–14 μm_ ∼ 0.21) throughout the entire infrared region. This is very effective in suppressing infrared thermal radiation. The dual‐infrared detection band thermal radiation channel of MRE switches from “open” to “closed” with dynamic phase transition, meeting the requirements of infrared detection in different situations. Furthermore, the emissivity spectrum of the MRE exhibits negligible variation as the temperature increases in Supporting Information S1: Figure S6. This indicates that the fabricated MRE maintains excellent thermal stability, which lays a solid foundation for its practical implementation.

Then, the high refractive index of ZnS can effectively modulate the VIS reflection spectrum by varying its thickness, which in turn contributes to the generation of a wide visible color gamut. Figure 3g presents the visible reflection spectra of the fabricated MRE with different ZnS layer thicknesses. For thickness variations of the top ZnS layer spanning 130–230 nm, the visible reflection spectrum of the MRE exhibits a pronounced redshift. Within the context of thin film interference theory, thicker ZnS layers translate to greater values of the optical path length, a phenomenon that causes the reflection spectrum to shift toward longer wavelengths. Figure 3h presents the (*x*, *y*) chromaticity coordinate distribution of the MRE in the CIE‐1931 color space for different ZnS thicknesses. The obtained color gamut ranges from *x*: 0.17–0.39 and *y*: 0.17–0.44, covering a wide range of chromatic intervals. Optical images of the MRE with different top ZnS layer thicknesses vividly demonstrate the substantial color change of the MRE in the VIS band (Figure 3h). For the top ZnS layer, its specific thickness properties exclusively dictate the apparent color of the MRE within the visible spectrum. Notably, tuning the thickness of the top ZnS layer yields an insignificant impact upon the infrared‐band absorption spectrum exhibited by the MRE in Supporting Information S1: Figure S7. This precisely controllable color‐tunable characteristic allows the MRE to achieve visible camouflage that blends seamlessly with the background, while simultaneously facilitating the encoding of anticounterfeiting information through colorimetric schemes.

To visually verify the switchable infrared performance of the MRE, MWIR and LWIR cameras are utilized to capture its corresponding infrared images. The MRE and the reference objects (a high‐emissivity SiO_2_ film with *ε*_3–14 μm_ ∼ 0.9 and a low‐emissivity Cr film with *ε*_3–14 μm_ ∼ 0.3) are placed on a temperature‐controlled metal platform and heated simultaneously. When the heating temperature is set at 140°C, MRE(a) shows a radiation temperature 19.6% lower than that of the high‐emissivity SiO_2_ film (100.3°C) in Figure 3i. In contrast, when switching to the crystalline state, the radiation temperature of MRE(c) reaches 51.4°C, which is closely consistent with that of the low‐emissivity Cr film (57.6°C). Therefore, the thermal radiation signal of the MRE(c) is significantly suppressed, effectively reducing the infrared signature. By leveraging this characteristic, the “display” and “erasure” of infrared‐encoded information can be achieved. In the amorphous state, the MRE(a) enables infrared cameras to capture the encoded information (display). Conversely, when MRE(a) switches to the crystalline state, the encoded information becomes concealed (erasure). Furthermore, the MRE demonstrates similar emissivity switching behavior within the MWIR band (Supporting Information S1: Figure S10). The above results highlight the MRE's infrared dual‐band thermal radiation switching capability and thermal stability, further validating its adaptability to complex thermal environments.

### 2.5 Dual‐Band Anti‐Counterfeiting Response and Angle Insensitivity Verification

The prevalence of counterfeit labels presents a significant threat to public consumer safety, a challenge that is particularly acute in the art industry and related sectors. Among current mainstream anticounterfeiting strategies, single‐mode visible anticounterfeiting labels and single‐variable infrared anticounterfeiting labels have limited effectiveness [1, 44]. These methods are generally vulnerable to being replicated and frequently fail to achieve the required levels of detection accuracy. As counterfeiting methodologies continue to advance, the rational design and optimization of anticounterfeiting technologies that can integrate multiple information into a single system has become a critical challenge that the industry must address urgently. The MRE exhibits dual‐band response characteristics with mutual independence between the visible and infrared bands, which overcomes the functional limitations of conventional anticounterfeiting technologies. This development provides more robust technical support for the anticounterfeiting sector, greatly enhancing both the practical value and competitiveness of these technologies in real‐world applications.

To experimentally validate the dual‐information encryption design [45], the MRE was fabricated following the procedure shown in Supporting Information S1: Figure S11. A 200 nm Al layer was first deposited on a quartz glass substrate via electron beam evaporation to form the base layer. Using mask 1 for region‐selective masking, a 320 nm Ge layer and a 250 nm SiO_2_ layer were sequentially deposited via electron beam evaporation, thereby realizing spatially programmable encoding of the infrared channel (e.g., HNU, pattern code, and digital codes). Subsequently, mask 1 was removed and a 220 nm IST phase‐change film was deposited by magnetron sputtering. By modulating the phase transition of the IST film, the opening and closing of infrared channels were realized. Finally, visible information was encoded using mask 2 by depositing ZnS layers with varying thicknesses.

To visually demonstrate the dual‐band application potential of the MRE's anticounterfeiting potential for valuable items, we devise a practical verification scenario centered on art authentication. As illustrated in Figure 4a, the painting “Pine” is chosen as the experimental verification subject. An anticounterfeiting label integrating the MRE is embedded in the artwork to set up a “dual‐verification” anticounterfeiting mechanism. Initially, authenticity is verified through visible patterns. Subsequently, more precise verification is carried out using an infrared camera, thereby establishing a multiple anti‐counterfeiting. A visible anticounterfeiting label with a pine tree motif is located in the lower right corner of the painting in Figure 4b. For comparison, a reference sample (high emissivity) that lacks infrared switching capability is also fabricated in Supporting Information S1: Figure S12. Figure 4c demonstrates that flexible visible color coding can be realized by adjusting the thickness of the top ZnS layer, whereas the corresponding infrared coding characteristics can be manipulated by employing different masks. The yellow sample (top ZnS thickness: 130 nm) matches the color of yellow grass, the blue sample (top ZnS thickness: 190 nm) corresponds to the blue sky, and the green sample (top ZnS thickness: 230 nm) is consistent with the green pine pattern. It is utilized to encode anticounterfeiting information via different colors, thereby accomplishing the initial encryption of the painting. However, relying solely on visual recognition is insufficient to fully dispel concerns regarding authenticity. Therefore, an infrared switching mechanism for “displaying‐erasing” anti‐counterfeiting information has been implemented. When the MRE(a) is in the “display” mode, LWIR camera can clearly capture the rabbit motif, along with the “010” and “HNU” inscriptions within the label region. These features exhibit significant temperature differences compared to the surrounding environment, thus appearing as bright areas and facilitating the retrieval of infrared‐encoded information. When the MRE(c) is in the “erasing” mode, the radiation temperature of the label pattern approaches that of the surrounding environment. Consequently, the pattern becomes completely undetectable in the infrared imaging, effectively hiding the encoded infrared information. This infrared “display‐erasure” switching capability significantly improves the level of security and the anticounterfeiting reliability associated with the encoded information. In addition, high‐energy pulsed laser irradiation can be employed to achieve switching between the erase and display modes [46, 47]. Through these multiple validations, the authenticity of the painting can be verified with high precision.

**FIGURE 4:**
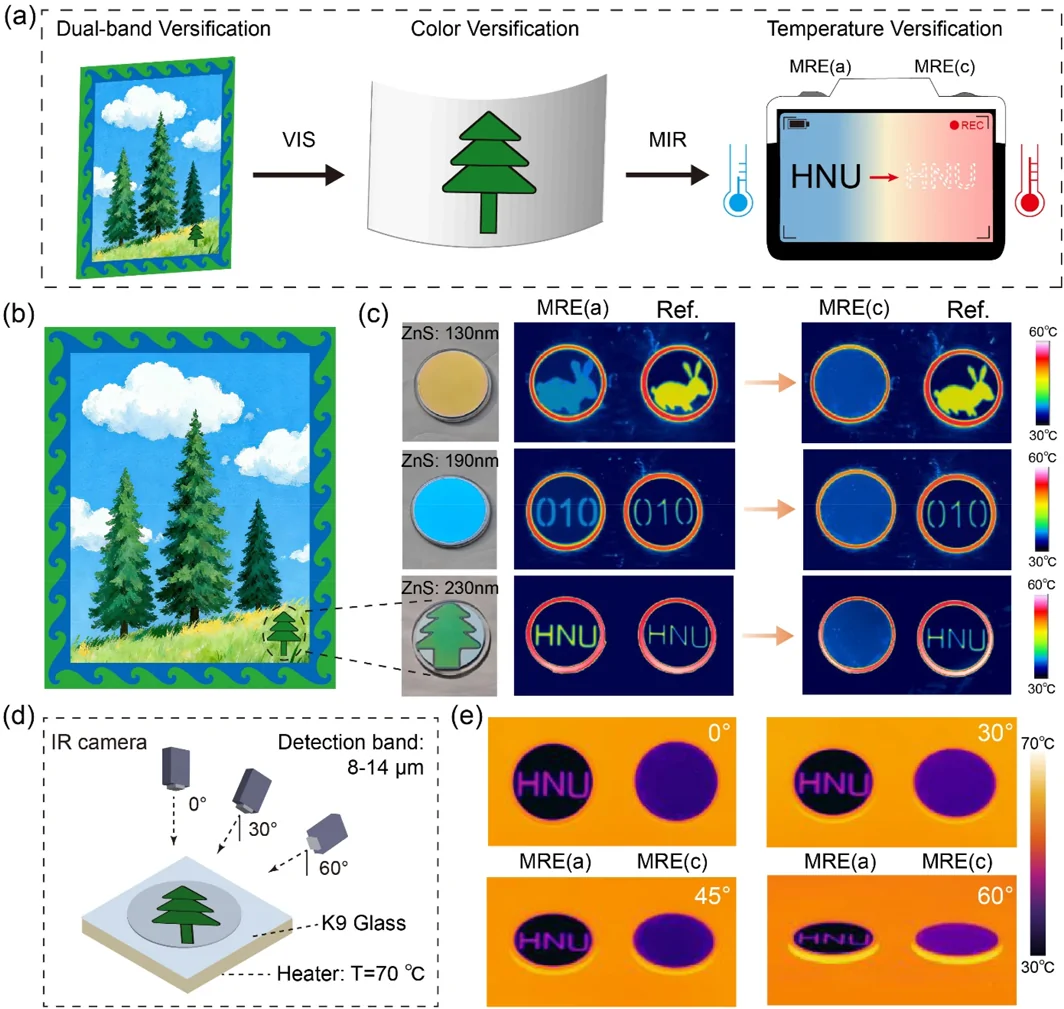
The dual‐band anticounterfeiting application and angle insensitivity verification of the MRE. (a) Schematic diagram of dual‐band anticounterfeiting verification. (b) The application of MRE as an anticounterfeiting label in the painting “Pine”. (c) Different types of anticounterfeiting encryption information images of the MRE. (d) Schematic diagram of infrared imaging devices from different angles. (e) LWIR thermal image of MRE (amorphous and crystalline states) is observed from four observation angles (0°, 30°, 45°, and 60°) at the heating temperature of 70°C.

Given the potential requirement for wide‐angle observation in practical detection, LWIR cameras are deployed to record infrared thermal images of the MRE at various detection angles. Figure 4d illustrates a schematic diagram of the experimental setup. Regardless of the detection angle in Figure 4e, the “HNU” pattern in MRE(a) remains easily distinguishable, whereas MRE(c) consistently presents an “invisible” state within the entire observation range. Furthermore, the infrared switching characteristics of MRE remain effective even at an observation angle as wide as 60°. Corresponding emissivity spectrum measurements further confirm that modifications to the incident angle produce a negligible influence on the infrared emission spectrum of the MRE in Supporting Information S1: Figure S13. These results indicate that the MRE can reliably realize infrared switchable “display‐erasure” functionality across a wide angular range of detection orientations, making it suitable for application in complex real‐world detection scenarios.

## 3 Conclusions

In summary, we present a novel multispectral response emitter that demonstrates adaptive dual‐band spectral response capabilities in both the visible and infrared regions. Through meticulous photonic structural design, the emitter enables precise modulation of the visible reflection spectrum. By leveraging a unique infrared capture mechanism combined with photonic amplification and the phase transition characteristics of the IST material (transitioning from a “lossless dielectric” to a “lossy metal”), the MRE achieves dynamic infrared switching between high and low emissivity states in both the MWIR and LWIR bands. Tuning of the visible spectrum yields an insignificant impact upon the infrared spectral domain, a feature that ensures the encrypted information in the infrared region is fully obscured by the visible signal. This method establishes a multiple information protection mechanism that consists of both visual response and infrared response. From a manufacturing point of view, the MRE employs a simple multilayer film stacking structure. It can be applied to both rigid substrates and flexible surfaces as long as the supporting material can endure the phase transition temperature of 300°C. This characteristic significantly enhances the flexibility of its deployment in practical applications. Overall, this multiband collaborative regulation capability surpasses the functional limitations of traditional single‐band devices and provides a novel pathway for the advancement of metamaterial technologies.

## 4 Experimental Section

*Numerical Simulations:* The emission properties and electromagnetic distribution of the emitter are numerically simulated using the RF module (electromagnetic waves and frequency domain) of COMSOL Multiphysics. Periodic boundary conditions are imposed on the simulation domain in the ± *x* directions, whereas the ± *z* directions are truncated with perfectly matched layer (PML) boundaries. An x‐polarized plane wave propagating along the negative *z*‐axis is utilized as the excitation source for the entire simulation framework.

*Fabrication of the MRE:* Firstly, a 1 mm thick SiO_2_ substrate is thoroughly cleaned using acetone and then subjected to ultrasonic treatment for 5 min. Subsequently, the SiO_2_ substrate undergoes sonication‐assisted cleaning with ethanol and deionized water to eliminate the organic matter on its surface, guaranteeing its complete cleanliness. Following this, all individual film layers are deposited via an electron beam evaporation system. The respective deposition rates for the target materials were set as follows: Al at 2 Å/s, Ge at 0.8 Å/s, and SiO_2_ at 0.7 Å/s. Given the complexity of the process involved in preparing IST film, a magnetron sputtering deposition system is used to sputter In_3_SbTe_2_ targets at a rate of 1 Å/s (where 10 Å = 1 nm) to obtain 220 nm IST films. The top ZnS layer is added using an electron beam evaporation system (0.8 Å/s) to fabricate the emitter. Ultimately, film layers with the following thicknesses are achieved: Al at 200 nm, Ge at 320 nm, SiO_2_ at 250 nm, IST at 220 nm, and ZnS at 230 nm. The fabricated emitter is placed in the GSL 1200X tube furnace and annealed at 300°C for 10 min under vacuum conditions to obtain crystalline emitter.

*Characterization of the emitter:* The infrared images are captured by using MWIR camera (IRMC‐615 MW, 3.8–4.7 μm, and IRSV) and LWIR camera (288+, 7–14 μm, and FOTRIC) to monitor the radiative temperature of the emitter across a range of heating temperatures. The SEM images of the emitter are obtained through the field emitted scanning electron microscopy (FESEM) (SUPRA40, Zeiss). The X‐ray diffraction analysis of the emitter is obtained using a diffractometer (D‐8 Discovery, Bruker). The atomic force microscope (MultiMode 8, Bruker) is used to measure the surface roughness and morphology of the film. The visible spectra of the MRE are measured using an ultraviolet‐visible‐near‐infrared spectrophotometer (Cary 5000). The reflectance spectra of the MRE are measured using Fourier transform infrared (FTIR) spectrometer (Bruker 70V).

## Author Contributions

**Jinlong Huang:** writing – original draft, formal analysis. **Xiuyu Chen:** investigation, methodology, validation. **Shuwen Zheng:** data curation. **Kun Yu:** funding acquisition, project administration. **Mengdan Qian:** conceptualization, supervision. **Yufang Liu:** writing – review and editing, resources.

## Supporting information


Supporting Information S1

## Data Availability

The data that support the findings of this study are available in the Supporting Information S1 of this article.
