# Supplementary material for: Non‐Volatile In3SbTe2 Multispectral Responsive Emitter With Visible Color and Infrared Display‐Erasure Function
Source: Nanophotonics. 2026 Apr 16;15(8):e70084. doi: 10.1002/nap2.70084 (PMC13642097; doi:10.1002/nap2.70084)
Supplement: Supplementary file 1 [file NAP2-15-e70084-s001.docx]

Supporting Information

Non-volatile In_3_SbTe_2_ Multispectral Responsive Emitter with Visible Color and Infrared Display-Erasure Function

Jinlong Huang, Xiuyu Chen, Shuwen Zheng, Kun Yu, Mengdan Qian*, and Yufang Liu*

**Figure S1.** Refractive index of IST in (a) the amorphous state and (b) the crystalline state [1].

The IST film exhibits high transparency to infrared radiation in its amorphous state. For the MRE in the amorphous state, the resonance peak located at 4.17 μm arises from the higher-order standing wave resonances supported by the stacked dielectric layers consisting of Ge, SiO_2_, IST, and ZnS. Meanwhile, the resonance peak at 9.36 μm originates from the first‑order standing wave resonance excited by the cooperative response of the entire dielectric stack (Ge, SiO_2_, IST, and ZnS). The power loss reveals that the loss at the 4.17 μm resonance is dominantly localized within the Al layer. In contrast, at the 9.36 μm resonance, substantial optical loss appears not only in the Al layer but also in the SiO_2_ layer. When the MRE is switched to the crystalline state, the overall optical loss becomes primarily concentrated within the IST layer. This result further confirms that the IST film in its crystalline phase is opaque to infrared radiation.

**Figure S2.** The normalized electric field |E| and power loss |Q| of MRE are respectively observed at wavelengths of 4.17 μm and 9.36 μm.

**Figure S3.** (a, b) Simulate the influence of varying the thickness of SiO_2_ on the absorption spectrum of MRE (amorphous and crystalline states). (c, d) Simulate the influence of varying the thickness of Ge on the absorption spectrum of MRE (amorphous and crystalline states).

**Figure S4.** Different scale sections of MRE.

**Figure S5**. EDX elemental mapping of the sample.

**Figure S6.** (a, b) Measured emissivity spectra of MRE at different temperatures.

**Figure S7.** (a, b) Simulate the influence of changing the top ZnS thickness on the MRE (amorphous and crystalline states) infrared absorption spectrum. (c, d) Measure the influence of changing the thickness of the top ZnS layer on the MRE (amorphous and crystalline states) infrared absorption spectrum.

To investigate the angular dependence of the visible structural color, we measured the color evolution of a 230 nm-thick top ZnS layer at varying incident angles. CIE 1931 chromaticity coordinate analysis reveals that the visible structural color varies with incident angles ranging from 15° to 45°. For the 230 nm-thick film, it exhibits a light-green color at 15°, transitions to bluish-green at 30°, and shifts to blue-green at 45°. Overall, the visible structural color exhibits weak angular dependence.

**Figure S8.** Variation of visible structural color with incident angle.

**Figure S9.** (a) The radiation temperatures of MRE and references (SiO_2_ and Cr) at different heating temperatures. (b) Measured infrared emission spectrum of the references (Cr and SiO_2_).

**Figure S10.** (a) The MWIR radiation temperatures of MRE and references (SiO_2_ and Cr) at different heating temperatures. (b) Thermal image at the heating temperature of 120 ℃.

**Figure S11.** The dual encoded information preparation process of MRE.

**Figure S12.** Infrared emissivity spectra of reference substances lacking switching function.

**Figure S13.** (a, b) Measured infrared absorption spectra of MRE (amorphous and crystalline states) at different incident angles.

**Reference**

1.	W.Y. Zha, Y.N. Zhu, B.Z. Ma, J.B. Yu, P. Ghosh, M. Qiu, and Q. Li, “Nonvolatile High-Contrast Whole Long-Wave Infrared Emissivity Switching Based on In_3_SbTe_2_,” *ACS Photonics* 10, no. 7, (2022): 2165-2172. <https://doi.org/10.1021/acsphotonics.2c00714>
